# Phylogenetic Reconstruction and Divergence Time Estimation of *Blumea* DC. (Asteraceae: Inuleae) in China Based on nrDNA ITS and cpDNA *trnL-F* Sequences

**DOI:** 10.3390/plants8070210

**Published:** 2019-07-08

**Authors:** Ying-bo Zhang, Yuan Yuan, Yu-xin Pang, Fu-lai Yu, Chao Yuan, Dan Wang, Xuan Hu

**Affiliations:** 1 Tropical Crops Genetic Resources Institute/Hainan Provincial Engineering Research Center for Blumea Balsamifera, Chinese Academy of Tropical Agricultural Sciences (CATAS), Haikou 571101, China; 2 School of Traditional Chinese Medicine Resources, Guangdong Pharmaceutical University, Guangzhou 510006, China

**Keywords:** *Blumea* DC., cpDNA *trnL-F*, diversification time, nrDNA ITS, temporal origin

## Abstract

The genus *Blumea* is one of the most economically important genera of Inuleae (Asteraceae) in China. It is particularly diverse in South China, where 30 species are found, more than half of which are used as herbal medicines or in the chemical industry. However, little is known regarding the phylogenetic relationships and molecular evolution of this genus in China. We used nuclear ribosomal DNA (nrDNA) internal transcribed spacer (ITS) and chloroplast DNA (cpDNA) *trnL-F* sequences to reconstruct the phylogenetic relationship and estimate the divergence time of *Blumea* in China. The results indicated that the genus *Blumea* is monophyletic and it could be divided into two clades that differ with respect to the habitat, morphology, chromosome type, and chemical composition of their members. The divergence time of *Blumea* was estimated based on the two root times of *Asteraceae*. The results indicated that the root age of Asteraceae of 76–66 Ma may maintain relatively accurate divergence time estimation for *Blumea*, and *Blumea* might had diverged around 49.00–18.43 Ma. This common ancestor had an explosive expansion during the Oligocene and Miocene and two major clades were differentiated during these epochs 29.60 Ma (17.76–45.23 Ma 95% HPD (Highest Posterior Density). Evidence from paleogeography and paleoclimate studies has confirmed that *Blumea* experienced differentiation and an explosive expansion during the Oligocene and Miocene.

## 1. Introduction

The genus *Blumea* is one of the largest genera of Inuleae (Asteraceae), containing approximately 100 species [1,2,3]. It is most diverse in Asia, Africa, and Australia, and it has more than one main center of diversity in Africa and South Asia [3]. China is one center of diversity of this genus, with 30 species being distributed throughout South China, of which five are endemic [4,5]. *Blumea* DC. has economic and ecological value in China. More than half of the species that belong to this genus have medical or ethnobotanical value [6]; for example, *Blumea balsamifera* (L.) DC. is an economic source of L-borneol or camphor extraction, and it is widely cultivated in the Philippines and China [7,8,9]. *Blumea megacephala* (Randeria) Chang et Tseng and *Blumea riparia* (Bl.) DC. are important sources of medical materials for “fuxuekang” [8]. Other species, such as *Blumea aromatica* DC., *Blumea formosana* Kitam, and *Blumea densiflora* DC., are used to treat rheumatism, esophagitis, headache, and hypertension [6]. Zhang and Cheng first described the taxonomy, plant morphology, and anatomy of the genus *Blumea* in China [4]; the authors treated six subsections and 30 species. Since the first description of this genus, its phylogenetic analysis and molecular evolution have been vigorously disputed. Phylogenetic analysis using molecular markers has been widely used to attempt to resolve this dispute; Pornpongrungrueng conducted important research [10,11], whose results showed that *Blumea* is monophyletic if the genera *Blumeopsis* Gagnep. and *Merrittia* Merr. are included, and suggested that this genus could be divided into two main clades that differ with respect to habitat, ecology, and distribution; however, few samples of the *Blumea* species from China were included [10,11]. In this study, we extended the molecular phylogenetic analysis of *Blumea* by (1) adding several samples from China to provide more thorough coverage of the overall distribution and diversity of *Blumea*, and (2) performing divergence time estimation. We aimed to: (1) estimate the molecular evolution and phylogenetic relationships of *Blumea* with a focus on the genus in China and (2) offer the best hypothesis for the divergence time and evolutionary events of this genus in China.

## 2. Results

### 2.1. Features of nrDNA ITS and cpDNA trnL-F Sequences in Blumea DC.

Based on the results of the sequence features and substitution models of single sequences or combined sequences from MEGA 7.0 [12], DAMBE [13], and jmodeltest 2.1.7 [14], the results indicated (Table 1): (1) the nrDNA ITS sequence ranged from 694 to 738 bp; after alignment, the sequence contained 657 characters, including 230 constant characters (35.01%), 314 informative characters (47.79%), and 114 uninformative characters (17.20%). (2) The chloroplast DNA (cpDNA) *trnL-trnF* intergenic spacer sequence ranged from 766 to 850 bp; after alignment, the sequence contained 858 characters, which included 730 constant characters (85.08%), 58 informative characters (6.76%), and 70 uninformative characters (8.16%). (3) The combined sequence ranged from 1,423 to 1,507 bp, including 960 constant characters (63.49%) and 370 informative characters (24.47%). The results from the incongruence length difference (ILD) test with PAUP 4.0a164 [15] indicated that the nrDNA ITS sequence and cpDNA *trnL-F* sequence could be combined (*p* = 0.19).

The results of the best substitution models from jmodeltest 2.1.7 with Akaike information criterion (AIC) [16] and Bayesian information criterion (BIC) [17] indicated that the best substitution models for ITS, *trnL-F*, or combined sequnces were, respectively, as SYM (symmetrical model) + I (proportion of invariable sites) + G (gamma distribution), TVM (transversion model) + G, and GTR (general time reversible) + I + G (Table 1); Table 1 also lists other parameters.

### 2.2. Phylogenetic Relationship of Blumea DC.

**Figure 1:**
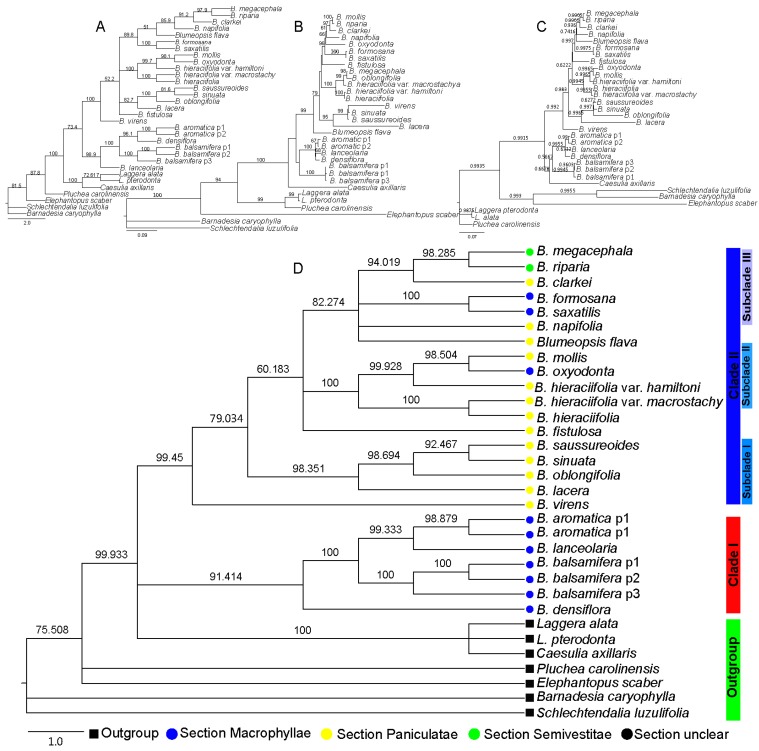
Phylogenetic tree based on the nrDNA internal transcribed spacer (ITS) sequence, where the numbers above the branches indicate the bootstrap value or posterior probabilities. (**A**) Phylogenetic tree determined by Neighbor-joining (NJ) method using the PAUP program; the numbers above the branches indicate the bootstrap value. (**B**) Phylogenetic tree with best topology, determined by Maximum Parsimony (MP) method using the PAUP program. The numbers above the branches indicate the bootstrap value. (**C**) Phylogenetic tree determined by Bayesian inference (BI) method using the MrBayes program; the numbers above the branches indicate the posterior probabilities. (**D**) Phylogenetic tree determined by Maximum Likelihood (ML) method using the IQtree program; the numbers above the branches indicate the bootstrap value.

Based on the sequence of nrDNA ITS, with four different phylogenetic analysis methods—Neighbor-joining (NJ), Maximum Parsimony (MP), Maximum Likelihood (ML), and Bayesian inference (BI)—the results of all indicated that the *Blumea* genus was monophyletic, but the topology of the four methods was different. For example, the topology of the NJ method (Figure 1A) indicated that *Blumea* could be divided into two clades. The phylogenetic relationship of the *B. aromatica* clade may be clear, but the phylogenetic relationships of the others were disordered, also with very low posterior probabilities. The results from the MP method (Figure 1B) seem to mostly conform to the traditional classification relationship of *Blumea*; the genus could be divided into three clades: clade *B. balsamifera* was the basal clade, the *B. aromatica* clade may be the transitional clade, and others may be the higher group with more extensive environmental adaptability, such as with one-year lifetime, diverse habitats, and morphological characteristics. The results of the BI method (Figure 1C) seem to be the same as those of the NJ method; also, with high posterior probabilities, but *Caesulia axillaris* Roxb is placed in the *B. aromatica* clade. The topology with different methods was compared with TreePuzzle 5.3.rc16 [18] and CONSEL [19], and the results indicated that the ML method was the most suitable tree-reconstruction method for ITS sequences and with first rank (Table 2). The genus of Blumea could be divided into two major clades based on the phylogenetic tree with ITS sequence and the MP method (Figure 1D). Clade I consisted of three species of Macrophyllae (including *B. aromatica*, *B. densiflora*, and *B. balsamifera*) and one species of section Paniculatae (*B. lanceolaria* (Roxb.) Druce.), but with a very low bootstrap. Clade II, with a perfect bootstrap, consisted of three subclades and two single species, *B. virens* DC. and *B. fistulosa*. Subclade I consisted of three species of section Paniculatae (including *B. saussureoides* Chang & Tseng, *B. sinuata* (Lour.) Merr., *B. oblongifolia* Kitam, and *B. lacera (Burm. f.) DC.*), with an ideal bootstrap value. Subclade II consisted of three species (*B. oxyodonta* DC., *B. mollis* (Burm. f.) DC., and *B. hieraciifolia* (D. Don) DC.) and two variants of *B. hieraciifolia* (*B. hieraciifolia* var. *hamiltoni* and *B. hieraciifolia* var. *macrostachy*). Subclade III consisted of seven species, but with diverse inscape, which include two species belong to Semivestitae section (*B. megacephala* and *B. riparia*), two species belong to Macrophyllae section (*B. formosana* and *B. saxatilis* Zoll. & Mor.), two species belong to Paniculatae section (*B. napifolia* DC. and *B. clarkei* Hook. f.), and *Blumeopsis flava* Gagnep.

**Figure 2:**
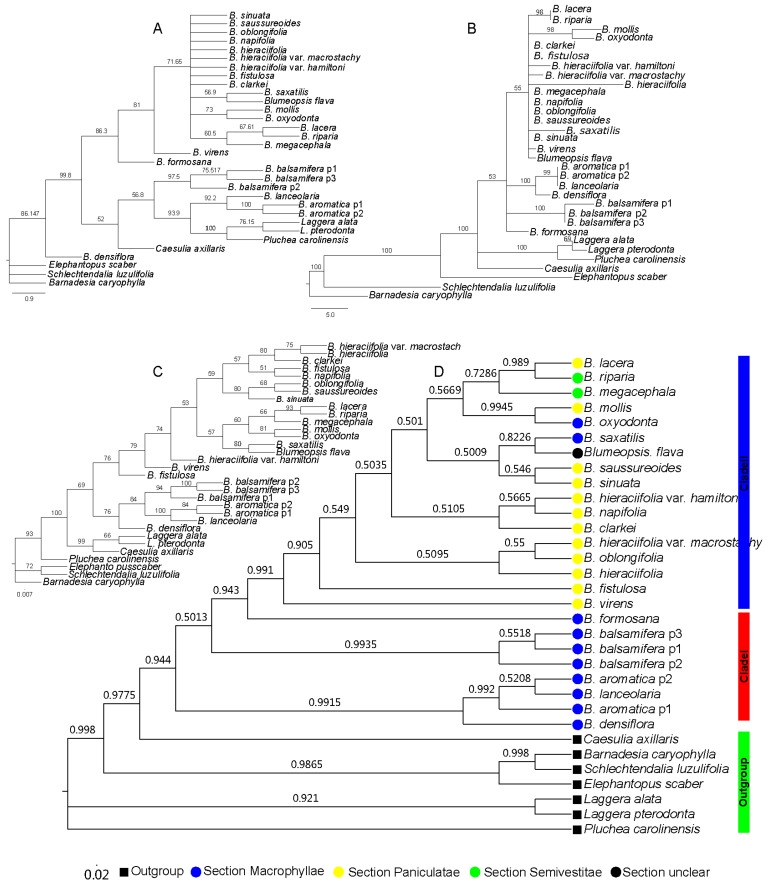
Phylogenetic tree based on the cpDNA trnL-F sequence, where the numbers above the branches indicate the bootstrap value or posterior probabilities. (**A**) Phylogenetic tree determined by the NJ method using the PAUP program; the numbers above the branches indicate the bootstrap value. (**B**) Phylogenetic tree with best topology, determined by the MP method using the PAUP program; the numbers above the branches indicate the bootstrap value. (**C**) Phylogenetic tree determined by the ML method using the IQtree program; the numbers above the branches indicate the bootstrap value. (**D**) Phylogenetic tree determined by the BI method with using the MrBayes program; the numbers above the branches indicate the posterior probabilities.

With the cpDNA *trnL-F* sequence, the results of the four different methods also indicated that the genus Blumea was divided into two clades, but the relationships that were revealed by the four methods were different, especially for the species in clade II (Figure 2). Combining the topology results indicated that the Bayesian inference (BI) method was possibly the most suitable tree reconstruction method (Table 2). With the BI method, Blumea could also been divided into two clades: clade I, with the same members of the nrDNA ITS dataset, consisting of three species of Macrophyllae (*B. aromatica*, *B. densiflora*, and *B. balsamifera*) and one species of section Paniculatae (*B. lanceolaria*), and the other species were combined into clade II, but with low posterior probabilities (Figure 2D). The same results were observed with the NJ, MP, and ME methods (Figure 2A–C): the genus could be divided into two clades, but with a low bootstrap value, especially in the phylogenetic tree in the NJ and MP methods. The scant information in the *trnL-F* dataset may have caused this.

**Figure 3:**
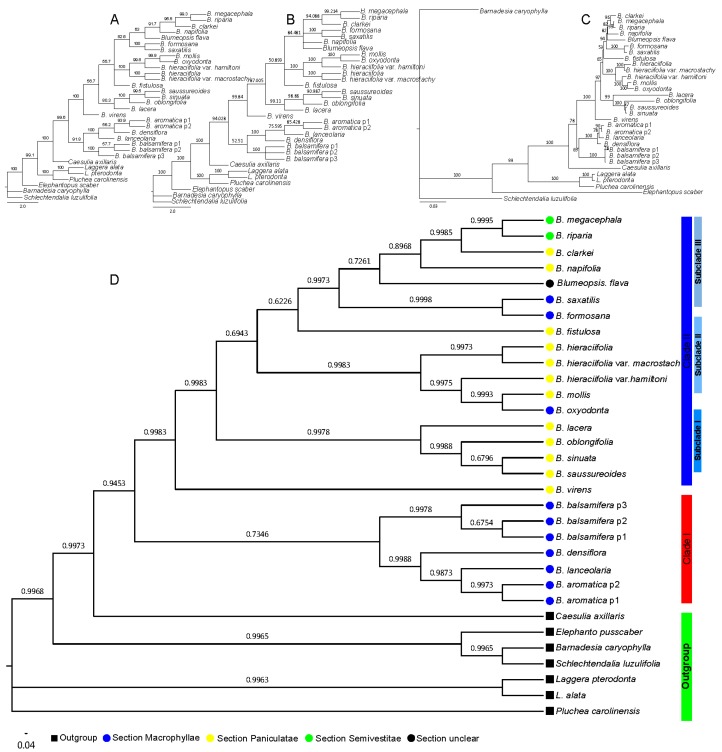
Phylogenetic tree based on the combined sequence; the numbers above the branches indicate the bootstrap value or posterior probabilities. The numbers above the branches indicate the bootstrap value. (**A**) Phylogenetic tree determined by the neighbor-joining (NJ) method using the PAUP program, (**B**) phylogenetic tree determined by the MP method using the PAUP program, (**C**) phylogenetic tree determined by the ML method using the IQtree program, and (**D**) phylogenetic tree determined by the BI method using the MrBayes program.

Furthermore, the results of the combined sequences indicated that the BI method was also the most suitable tree-reconstructed method for combined sequences (Table 2), with the same topology based on the nrDNA ITS sequence when analyzed with all four methods, (Figure 3). The genus Blumea could be divided into two clades; clade II also consisted of three subclades and two single species (Figure 3D). The same thing was observed from the results of the NJ, MP, and ME methods (Figure 3A–C), and with a better bootstrap posterior supported value. This means that the combined sequence can improve the lower posterior probabilities or bootstrap that is caused by the single sequence.

### 2.3. Divergence Time Estimation and Evolutionary Event Hypothesis

**Figure 4:**
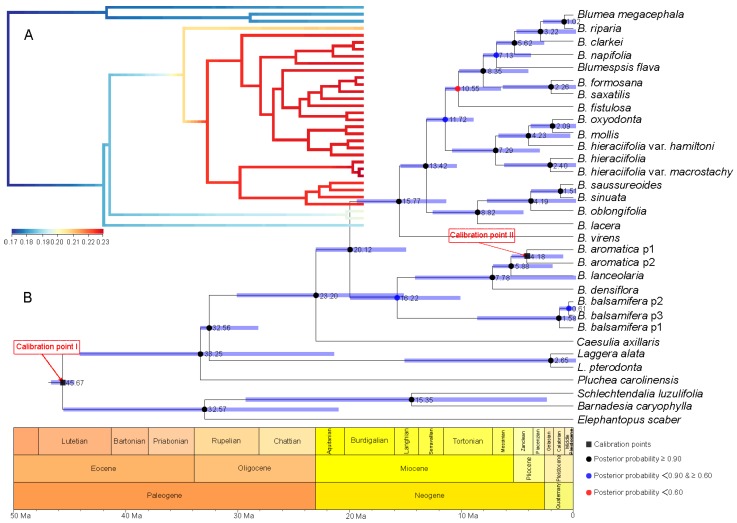
Divergence time estimation and speciation–extinction rate analysis based on the root age of Asteraceae as 49–42 Ma [20,21,22] and combined sequence. (**A**) The speciation–extinction rates analysis performed by BAMM [23] and BAMMtools [24]. (**B**) The divergence time estimation performed by BEAST 1.10.4 [25], and geological time scale visualized with the strap package [26]. The values beside nodes indicate the estimated median time of differentiation, and error bars indicate 95% highest posterior density (HPD) of differentiation. The black squares on nodes indicate calibration time. The circles with black, blue and red respectively indicate posterior probability more than 0.90, 0.60–0.90, and less than 0.60.

**Figure 5:**
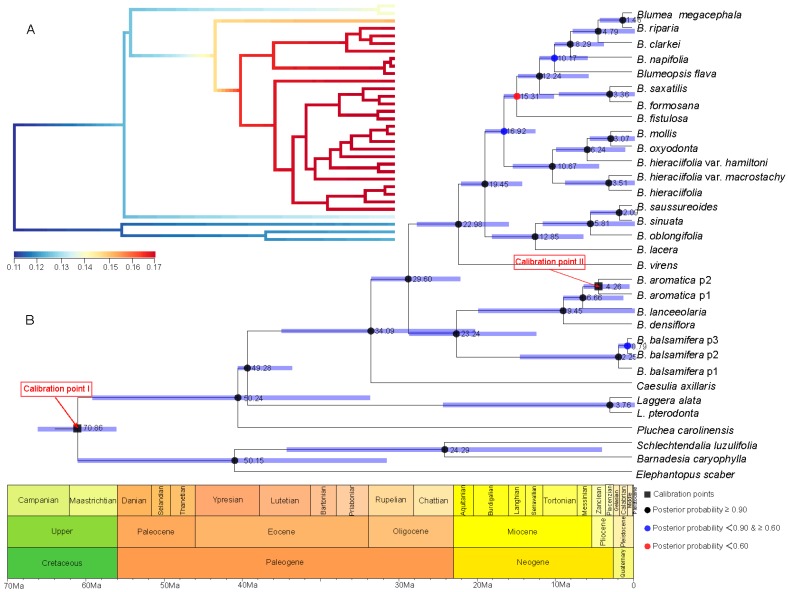
Divergence time estimation and speciation–extinction rates analysis based on the root age of Asteraceae as 76–66 Ma [27] and the combined sequence. (**A**) The speciation–extinction rates analysis performed by BAMM [23] and BAMMtools [24]. (**B**) The divergence time estimation performed by BEAST 1.10.4 [25], and geological time scale visualized with the strap package [26]. The values beside nodes indicate the estimated median time of differentiation, and error bars indicate 95% HPD of differentiation. The black squares on nodes indicate calibration time. The circles with black, blue and red respectively indicate posterior probability more than 0.90, 0.60-0.90, and less than 0.60.

A series of evolutionary events and divergence times were estimated based on a comparison of the two calibration points of the root age of Asteraceae and geography–species differentiation time (Figure 4 and Figure 5). With the root age of Asteraceae as 49–42 Ma [20,21,22] (Figure 4), the time of genus *Blumea* differentiated from Inuleae is estimated to be 23.20 Ma, with the highest posterior density (HPD) between 32.86 and 14.51 Ma (the time between the late Oligocene and the early Miocene). The divergence time of the two major clades in *Blumea* is estimated to be 20.12 Ma (with HPD between 28.89 and 12.40 Ma). Further investigation with BAMM [23] and BAMMtools [24] showed that this genus underwent an explosive expansion during the Miocene, and many new species speciate during this period (Figure 4A).

With the root age of Asteraceae as 76–66 Ma [27] (Figure 5), the time genus Blumea differentiated from Inuleae is estimated to be 34.09 Ma, with a HPD between 49.00 and 18.43 Ma (the time between the late Oligocene and the early Miocene), and the divergence time of the two major clades is estimated to be 45.23–17.76 Ma (median time of 29.60). Further investigation with BAMM [23] and BAMMtools [24] showed that (Figure 4A) this genus experienced an explosive expansion, since it differentiated from Inuleae.

**Figure 6:**
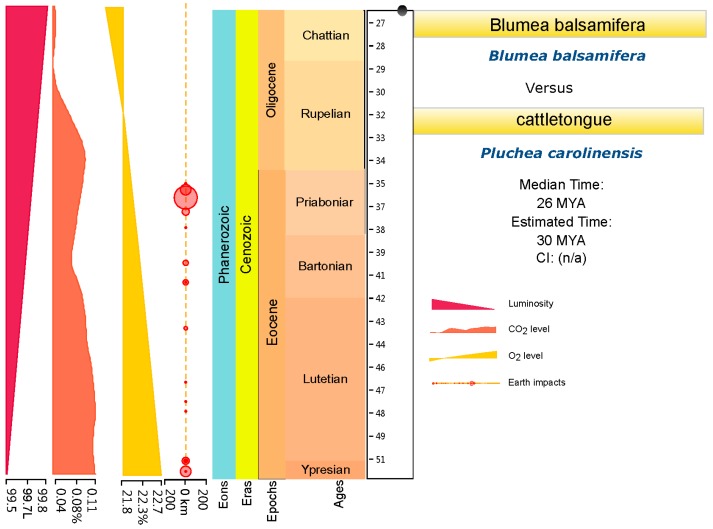
The divergence time estimation between *Blumea balsamifera* DC. and *Pluchea. Carolinensis* (Jacq.) G. Don., based on TimeTree.

Two series of divergence time were estimated with two different root ages of Asteraceae (76–66 Ma [27] and 49–42 Ma [20,21,22]) (Figure 4 and Figure 5). A third web tool, TimeTree (http://www.timetree.org/) [28], was used for further divergence time estimation and for making the final decision. The results indicated that the median divergence time of *B. balsamifera* and *Plumea carolinensis* (Jacq.) G. Don. may have been in the early Miocene, around 26 Ma ago (Figure 6). Combining the results of TimeTree with the two calibration points for the root age of Asteraceae, we think that the root age of Asteraceae of 76–66 Ma [27] may be relatively accurate divergence time estimation for *Blumea*. First, based on the paleogeography and paleoclimate of the Oligocene (Figure 6), the Oligocene interrupted the temperature decline in the Paleogene (~33.5 Ma ago), and a stepwise climate increase began at 32.5 Ma and lasted until 25.5 Ma ago [29,30], also accompanied with a reduction of the CO_2_/O_2_ rate. This same time was the most important period for grassland and forest expansion, and the sunflower family also underwent an explosive expansion during this time [27]. The early Miocene (~20 Ma ago) is the time of formation and differentiation of Malaysian flora [31]. The old Chinese mainland, because of the relatively warm climate, was the most fertile area on Earth, had become one diversity center of earth. The genus of *Blumea*, which mostly originated from palaeotropical region, had moved to south China, also accompanied with an explosive expansion for more herbal members, such as *B. saussureoides*, *B. sinuata*, *B. oblongifolia*, *B. lacera*, and others that are more suitable for the low CO2/O2 rate.

Combining the evidence from paleogeography and paleoclimate and two root ages of Asteraceae, the originate time of genus *Blumea* may be estimated to be 49.00–18.43 Ma; the divergence time of the two major clades was further estimated to be 45.23–17.76 Ma ago. This genus experienced an explosive expansion during the Oligocene and Miocene.

## 3. Discussion

The genus *Blumea* is one of the largest genera of Inuleae (Asteraceae), being widely distributed in ancient tropical regions around the Pacific. South China is one diversity center of this genus, containing 30 species. Given the variety of its morphology, its phylogenetic analysis and trait characterization have been vigorously disputed since the first description of this genus. Phylogenetic analysis using molecular markers has been widely used to attempt to resolve this dispute [10,11]. Pornpongrungrueng [11] conducted a phylogenetic analysis on *Blumea*. The results showed that *Blumea* (including *Blumeopsis flava*) is monophyletic, and suggested that this genus could be divided into two main clades and one single species, *B. balsamifera*, which differ in terms of habitat, ecology, and distribution. However, few samples were obtained from species in China. In this study, 16 Chinese samples, which include 12 species belonging to three sections of *Blumea*, that were sequenced with nrDNA ITS and cpDNA *trnL-F* sequences, were used to estimate the phylogenetic relationship between the members of this genus. The results repeatedly verified the conclusions of Pornpongrungrueng [11], which indicated that this genus could be divided into two clades: clade I, including *B. aromatica*, *B. densiflora*, *B. balsamifera*, and *B. lanceolaria*, and the others belonging to clade II. When comparing the habitat, distribution, chromosome number, and chemical composition, we found that the two main clades differ in all of these aspects plus ecology, with the Blumea clade mostly containing perennial shrubs or subshrubs, which were mostly distributed from evergreen forests, and those with the same chromosome number (2n = 18) and chromosome structure (6M + 8m + 4sm) [32], and whose chemical composition is similar [33]. Clade II is a widespread paleotropical group that comprises mostly annual, weedy herbs of open forests and fields. Divergence time estimation and evolutionary event hypothesizing are important tools in phylogenetic analysis, but, it is difficult to estimate evolutionary events and divergence time because of the fossil record. Choosing some geological events as calibration points could provide more evidence for the process of phylogenetic evolution and divergence event hypothesis [34]. Most of the families have no fossil record because the fossil record information on Inuleae is scant. Several fossils are clearly identifiable as members of Asteraceae and Goodeniaceae from the Oligocene and, later, and there are seeds of Menyanthaceae and Campanulaceae from the Oligocene and Miocene, respectively [35]. There are also several records of Eocene pollen of Asteraceae found in South America or China [36,37]. As Oligocene pollen of the Asteraceae is of a comparatively specialized type and it is found on several continents, it is reasonable to assume that it dates back at least to the Oligocene–Eocene boundary (42–49 Ma). Hind et al. estimated the same date [38]. However, according to a more recent paper by Barreda et al. [27], the most recent common ancestor of Asteraceae may have originated 76–66 Ma ago. In this research, by considering the time of formation of Hainan Island and Qiongzhou Strait (5.8–3.7 Ma) [39,40,41], we were able to estimate the divergence time of *Blumea* DC. The results indicated that *Blumea* may have originated from 49.00 to 18.43 Ma, and had an explosive expansion during the Oligocene and Miocene (45.23–17.76 Ma), when the two major clades differentiated. The evidence from paleogeography and paleoclimate are consistent with the conclusion that *Blumea* underwent differentiation and explosive expansion during the Oligocene and Miocene [29,30].

## 4. Materials and Methods

### 4.1. Plant Materials

In this study, 16 Chinese samples, including 12 species that belong to three sections of Blumea DC, were newly collected and sequenced, and the specimens of those samples were deposited in the herbarium of the Chinese Academy of Tropical Agricultural Sciences (herbarium code: CATCH). Nine reference samples of *Blumea* and seven samples of outgroups (seven species, six genera) were downloaded from Genbank. Table 3 provides the sequence information, locality, and other details of the accessions.

### 4.2. DNA Isolation

Genomic DNA was isolated from the above accessions while using the QIAGEN DNeasy Plant Minikit (QIAGEN, Düsseldorf, Germany), according to the manufacturer’s instructions. The DNA was diluted to 30 ng/μL and the ultraviolet absorption values at A_260_ were used.

### 4.3. DNA Amplification and Sequencing: ITS and trnL-F Sequence Amplification and Sequencing

Internal transcribed spacer (ITS) and *trnL-F* sequence amplification and analysis were conducted according to the previously established protocols [39,40]. The PCR mixture consisted of 60 ng DNA, 1.0 μM of each primer (Invitrogen Corp., Carlsbad, CA, USA), and 25.0 μL of 2× Taq PCR Master Mix (0.1 U/μL Taq polymerase, 500 μM of each dNTP, 20 mM Tris-HCl at pH 8.3, 100 mM KCl, 3 mM MgCl_2_; Tiangen, Beijing, China) in a 50-μL volume. The PCR cycle protocol was based on previously established methods [42]. The PCR products that were obtained were separated on a 1.2% agarose gel, and then the bands of the expected size were excised from the gel and purified while using the QIAquick Gel Extraction Kit (QIAGEN Inc., Valencia, CA, USA), according to the manufacturer’s instructions. The purified PCR products were subjected to sequencing (Invitrogen, Shanghai, China).

### 4.4. Sequence Processing, Conversion, and Analysis

#### 4.4.1. Single Sequence

First, the raw sequences of the ITS and *trnL-F* fragments (Invitrogen, Shanghai, China) were edited to remove the low-quality parts. The edited sequences were then submitted to GenBank (Table 3). The sequences of both loci were aligned while using MEGA 7.0 [12] with the default options and saved in *.nexus and *.aln formats. The interleaved *.nexus files were converted to the non-interleaved *.nex format while using PAUP 4.0a164 [15]. The sequence saturation and best substitution model were analyzed using DAMBE [13] and jmodeltest 2.1.7 [14], and then the poorly aligned positions and divergent regions in each aligned sequence were removed using Gblocks0.91b [43,44], using the filtration parameters that were described by Guillou [44].

#### 4.4.2. Combined Sequence

The datasets of ITS and *trnL-F* sequences were loaded into PAUP 4.0 (https://paup.phylosolutions.com/) [14] for compatibility testing (ILD test) [47]. The compatibility testing (ILD test) revealed no incongruence between ITS and trnL-F sequences (p = 0.19). Subsequently, the sequences were merged while using SequenceMatrix1.8 [48] and saved in *.nex format.

### 4.5. Phylogenetic Tree Construction

To reconstruct a more accurate phylogeny of *Blumea*, first, a single sequence or combined sequence was used for constructing a phylogeny tree while using four phylogenetic tree construction methods: neighbor-joining (NJ) method, the maximum parsimony (MP) method, the maximum likelihood (ML) method, and the Bayesian inference (BI) method in PAUP 4.0a164 [15], IQ-TREE [49,50], and MrBayes 3.2.6 [51]. Afterwards, the topology of the phylogenetic tree was evaluated with different methods in TreePuzzle 5.3.rc16 [18] and CONSEL software [19]. The phylogenetic trees with the best topology were edited with FigTree 1.4.2 (http://tree.bio.ed.ac.uk/software/figtree/) and Inkscape 0.9.2 (https://inkscape.org/).

#### 4.5.1. Neighbor-Joining (NJ) Method

The NJ analysis was performed with PAUP version 4.0a164 [15] with heuristic searches under the random option of the stepwise addition algorithm with equal weighting of all characters. Bootstrap 50% majority-rule consensus trees were reconstructed by performing heuristic searches with 1000 bootstrap replicates.

#### 4.5.2. Maximum Parsimony (MP) Method

The same as the NJ method, an MP analysis was reconstructed with PAUP 4.0a164 [15], performing heuristic searches with 1000 bootstrap replicates. 

#### 4.5.3. Maximum Likelihood (ML) Method

Before ML analysis with IQ-TREE [49,50], the best substitution models of the single or combined sequences were computed with jmodeltest 2.1.7 [14]. Subsequently, the phylogenetic tree with the ML method was analyzed with IQ-TREE [49,50] using the default settings, with 1000 bootstrap (BP) values for tree evaluation.

#### 4.5.4. Bayesian inference (BI) method

With the best substitution models and parameters that were computed by jmodeltest 2.1.7 [14], BI analysis was performed with MrBayes 3.2.6 [51], but not all of the substitution models computed by jmodeltest 2.1.7 [14] were available in MrBayes, so an General time reversible (GTR) model with unequal rates and unequal base freq) model were used for BI analysis, and the base frequency of the nucleic acids and transition/transversion rates were set according the results from jmodeltest 2.1.7 [14]. The analysis parameters were set as four chains that were run simultaneously for 10,000,000 generations or until the average standard deviation of the split frequencies fell below 0.01. The trees were sampled with every 100 generations and a total of 20,000 trees were generated with the initial sample. Subsequently, the consensus tree was summarized with LogCombiner 1.10.4 and TreeAnnotator 1.10.4, and the parameters were first set to 10%, but then discarded as burn-in, and a 50% majority-rule consensus tree with posterior probability (PP) values was used.

### 4.6. Divergence Time Estimation

#### 4.6.1. Time Node Selection and Correction

As there are no unequivocal fossils for *Blumea* DC and its relatives in Inuleae, the divergence and diversification times were difficult to estimate. Two root ages of Asteraceae were compared to accurately estimate divergence time estimation for *Blumea*, in this study: the recent results from Barreda et al. [27], who estimated the root age of Asteraceae at 76–66 Ma; and, the time generally accepted by other researchers, 49–42 Ma [20,21,22]. Subsequently, the geological ages of Qiongzhou Strait and Hainan Island, 5.8–3.7 Ma, were selected as the latest differentiation time of *B. aromatica* DC samples from Guizhou and Hainan [38,40,41]. To investigate the speciation rates of *Blumea* DC along with time, BAMM (http://bamm-project.org/) [23] and BAMMtools (https://cran.rstudio.com/web/packages/BAMMtools/index.html) [24] were used to compare the speciation rates with the two hypotheses of the root age of Asteraceae.

#### 4.6.2. Divergence Time Estimation and Evolutionary Event Hypothesis

All of the divergence times and evolutionary events were hypothesized while using BEAST 1.10.4 (http://beast.community/) [25]. First, the likelihood ratio test (LRT) [52] was used to determine that the ITS or trnL-F data conformed to the molecular clock hypothesis. The results indicated that the Blumea data were not suitable for the molecular clock hypothesis (p = 0.03). Afterwards, the divergence times were analyzed while using BEAST 1.10.4 [25], incorporating an uncorrelated lognormal clock model and a birth–death speciation process, and the nucleotide substitution model settings were compared with the jmodeltest results. The parameters were set to be similar to those used in MrBayes 3.2.6 during the analysis process [51], and with four gamma Categories, 10,000,000 generations for four independent Markov chain Monte Carlo (MCMC) runs, sampling every 10,000 generations, with substitution and clock models being unlinked between partitions. The convergence and effective sample size (>200) of parameters were analyzed with Tracer 1.7. LogCombiner 1.10.4 and TreeAnnotator 1.10.4 were used for summarizing the trees, and the parameters, which were set at first to 10%, were discarded as burn-in, and a 50% majority-rule consensus tree with PP values was used instead. Lastly, a strap package [26] in R was used for visualization of the results of BEAST to reflect the divergence time of *Blumea*, especially the geological time scale.

## Figures and Tables

**Table 1:** Features of Nuclear ribosomal DNA (nrDNA) internal transcribed spacer (ITS) and chloroplast DNA (cpDNA) tRNA gene L region (trnL)- tRNA gene F region (trnL) intergenic spacer sequences of *Blumea* DC.

DNA Region	Number of Characters	Number of Constant Characters	Number of Variable Characters	Number of Informative Characters	Percent of Informative Sites (%)	Best Substitution Model	Model Matrix			
							Rates	*N*cat	*P*-invar	Gamma Shape
nrDNA ITS	657	230	427	314	47.79	SYM + I + G ^a^	Gamma	4	0.2390	2.1770
cpDNA *trnL-F*	858	700	158	70	8.16	TVM + G ^b^	Gamma	4	0.0	0.9240
Combined sequence	1545	960	551	375	24.27	GTR + I + G ^a^	Gamma	4	0.0	0.7302

Notes: ^a^ SYM (symmetrical model) + I (proportion of invariable sites) + G (gamma distribution); ^b^ TVM (transversion model) + G; ^c^ GTR (general time reversible) + I + G.

**Table 2:** The topology compared with four phylogenetic tree construction methods.

DNA Region	Tree Method	Rank	obs^a^	au^b^	np^c^	bp^d^	pp^e^	kh^f^	sh^g^	wkh^h^	wsh^i^
nrDNA ITS	NJ^a^	3	2.6	0.358	0.331	0.095	0.067	0.337	0.557	0.285	0.635
	MP^b^	4	26.5	0.002	0.002	0.002	3.000 × 10^−12^	0.013	0.025	0.013	0.019
	ML^c^	1	−2.6	0.681	0.669	0.674	0.866	0.662	0.859	0.662	0.885
	BI^d^	2	2.6	0.357	0.331	0.229	0.067	0.338	0.558	0.338	0.648
cpDNA *trnL-F*	NJ	4	0.4	0.337	0.274	0.275	0.176	0.300	0.309	0.300	0.309
	MP	3	0.0	0.410	0.213	0.210	0.274	0.326	0.743	0.326	0.620
	ML	2	0.0	0.503	0.357	0.351	0.274	0.453	0.682	0.453	0.655
	BI	1	0.0	0.731	0.163	0.164	0.275	0.547	0.997	0.547	0.992
Combined sequence	NJ	4	8.5	0.097	0.077	0.077	1.000 × 10^−4^	0.125	0.229	0.125	0.187
	MP	3	0.0	0.328	0.293	0.301	0.009	0.297	0.435	0.297	0.413
	ML	2	0.0	0.165	0.055	0.056	0.495	0.085	0.753	0.085	0.691
	BI	1	0.0	0.867	0.578	0.566	0.496	0.915	0.982	0.915	0.986

Notes: NJ^a^ = Neighbor-joining; MP^b^ = Maximum Parsimony; ML^c^ = Maximum Likelihood; BI^d^ = Bayesian inference; Obs^a^ = observed log-likelihood difference to the best topology; au^b^ = approximately unbiased; np^c^ = bootstrap probability of the topology (i.e., the probability that the given topology has the largest likelihood in 10 scaled sets of 10,000 bootstrap replicates); bp^d^ = np with 10 non-scaled sets of 10,000 bootstrap replicates; pp^e^ = Bayesian posterior probabilities of the model; kh^f^ = Kishino–Hasegawa; sh^g^ = Shimodeira–Hasegawa; wkh^h^ = weighted Kishino–Hasegawa; wsh^i^ = weighted Shimodeira–Hasegawa.

**Table 3:** Localities and samples used in the present study.

Section	Code	Latin Name	Locality	GenBank Accession Number		Reference
				*ITS*	*trnL-F*	
**Semivestitae** **(2 spp.)**	*B. megacephala*	*Blumea megacephala* (Randeria) Chang & Tseng	Guangxi, China	KP052666	KP052682	This research
	*B. riparia*	*B. riparia* (Bl.) DC.	Yunnan, China	KP052668	KP052685	This research
**Macrophyllae** **(6 spp.)**	*B. balsamifera* p1	*B. balsamifera* (L.) DC.	Guizhou, China	KP052658	KP052674	This research
	*B. balsamifera* p2	*B. balsamifera* (L.) DC.	Yunnan, China	KP052659	KP052675	This research
	*B. balsamifera* p3	*B. balsamifera* (L.) DC.	Hainan, China	KP052660	KP052676	This research
	*B. aromatic* p1	*B. aromatica* DC.	Guizhou, China	KP052656	KP052672	This research
	*B. aromatic* p2	*B. aromatica* DC.	Hainan, China	KP052657	KP052673	This research
	*B. oxydonta*	*B. oxyodonta* DC.	Thailand	EU195665	EU195630	[11]
	*B. densiflora*	*B. densiflora* DC.	Thailand	EF210934	EF211029	[10]
	*B. saxatilis*	*B. saxatilis* Zoll. & Mor.	Australia	EF210945	EF211040	[10]
	*B. formosana*	*B. formosana* Kitam	Zhejiang, China	KP052665	KP052678	This research
	*B. clarkei*	*B. clarkei* Hook. f.	Thailand	EF210974	EF211069	[10]
**Paniculatae** **(9 spp. and 2 varieties)**	*B. fistulosa*	*B. fistulosa* (Roxb.) Kurz.	Yunnan, China	KP052661	KP052677	This research
	*B. hieraciifolia*	*B. hieraciifolia* (D. Don) DC.	Yunnan, China	KP052662	KP052679	This research
	*B. hieraciifolia* var. *hamiltonii*	*B. hieraciifolia* var. *hamiltonii*	Burma	EF210972	EF211067	[10]
	*B. hieraciifolia* var. *macrostachya*	*B. hieraciifolia* var. *macrostachya*	Thailand	EF210937	EF211032	[10]
	*B. lanceolaria*	*B. lanceolaria* (Roxb.) Druce.	Guangxi, China	KP052664	KP052681	This research
	*B. lacera*	*B. lacera* (Burm. f.) DC.	Zhejiang, China	KP052663	KP052680	This research
	*B. mollis*	*B. mollis* (D. Don) Merr.	Hainan, China	KP052670	KP052683	This research
	*B. napifolia*	*B. napifolia* DC.	Thailand	EF210959	EF211054	[10]
	*B. oblongifolia*	*B. oblongifolia* Kitam	Hainan, China	KP052667	KP052684	This research
	*B. saussureoides*	*B. saussureoides* Chang & Tseng	Yunan, China	KP052669	KP052686	This research
	*B. sinuata*	*B. sinuata* (Lour.) Merr.	Thailand	EF210948	EF211043	[10]
	*B. virens*	*B. virens* DC.	Thailand	EF210957	EF211052	[10]
**Uncertainty**	*Blumeposis flava*	*Blumeposis flava* Gagnep.	Thailand	EF210960	EF211055	[10]
**Outgroup**	*Caesulia axillaris*	*Caesulia axillaris* Roxb.	India	EF210949	EF211044	[10]
	*Pluchea carolinensis*	*Pluchea carolinensis* (Jacq.) G. Don.	Taiwan, China	AF437850	EU385104	[45]
	*Laggera alata*	*Laggera alata* (D. Don) Sch. Bip. ex Oliv.	Thailand	EF210930	EF211025	[10]
	*L. pterodonta*	*L. pterodonta* (DC.) Sch. Bip. ex Oliv.	Thailand	EF210929	EF211024	[10]
	*Schlechtendalia luzulifolia*	*Schlechtendalia luzulifolia*	Australia	KF989506	KF989612	[46]
	*Barnadesia caryophylla*	*Barnadesia caryophylla*	Australia	AY504686	AY504768	[46]
	*Elephantopus scaber*	*Elephantopus scaber* L.	Hainan, China	KP052671	KP052687	This research
